# Seasonal dynamics of soil microbiome in response to dry–wet alternation along the Jinsha River Dry-hot Valley

**DOI:** 10.1186/s12866-024-03662-1

**Published:** 2024-11-25

**Authors:** Hao Jiang, Xiaoqing Chen, Yongping Li, Jiangang Chen, Li Wei, Yuanbin Zhang

**Affiliations:** 1 Key Laboratory of Mountain Hazards and Earth Surface Processes, Institute of Mountain Hazards and Environment, Chinese Academy of Sciences, Chengdu, 610299 China; 2 State Key Laboratory of Mountain Hazards and Engineering Resilience, Institute of Mountain Hazards and Environment, Chinese Academy of Sciences, Chengdu, 610299 China; 3 School of Agriculture, Yunnan University, Kunming, 650500 China

**Keywords:** Altitudinal gradient, Dry-hot valley, Mountain-valley breeze circulation, Seasonal dry–wet cycle, Stochastic process

## Abstract

**Background:**

Soil microorganisms play a key role in nutrient cycling, carbon sequestration, and other important ecosystem processes, yet their response to seasonal dry–wet alternation remains poorly understood. Here, we collected 120 soil samples from dry-hot valleys (DHVs, ~ 1100 m a.s.l.), transition (~ 2000 m a.s.l.) and alpine zones (~ 3000 m a.s.l.) along the Jinsha River in southwest China during both wet and dry seasons. Our aims were to investigate the bacterial microbiome across these zones, with a specific focus on the difference between wet and dry seasons.

**Results:**

Despite seasonal variations, bacterial communities in DHVs exhibit resilience, maintaining consistent community richness, diversity, and coverage. This suggests that the microbes inhabiting DHVs have evolved adaptive mechanisms to withstand the extreme dry and hot conditions. In addition, we observed season-specific microbial clades in all sampling areas, highlighting their resilience to environmental fluctuations. Notably, we found similarities in microbial clades between soils from DHVs and the transition zones, including the phyla Actinomycetota, Chloroflexota, and Pseudomonadota. The neutral community model respectively explained a substantial proportion of the community variation in DHVs (87.7%), transition (81.4%) and alpine zones (81%), indicating that those were predominantly driven by stochastic processes. Our results showed that migration rates were higher in the dry season than in the wet season in both DHVs and the alpine zones, suggesting fewer diffusion constraints. However, this trend was reversed in the transition zones.

**Conclusions:**

Our findings contribute to a better understanding of how the soil microbiome responds to seasonal dry–wet alternation in the Jinsha River valley. These insights can be valuable for optimizing soil health and enhancing ecosystem resilience, particularly in dry-hot valleys, in the context of climate change.

**Supplementary Information:**

The online version contains supplementary material available at 10.1186/s12866-024-03662-1.

## Background

The soil microbiome, a diverse community of microorganisms, plays a pivotal role in ecosystem services such as nutrient cycling, carbon sequestration [1–4], and plant growth promotion [5]. However, its activities are sensitive to environmental factors including temperature fluctuations, precipitation patterns, and extreme events [6–10], potentially challenging soil microbial communities and ecosystem stability [11, 12]. Seasonal variations, particularly dry and wet alternations, significantly impact soil microbiome composition and function [4, 13, 14]. During the dry season, the soil microbiome experiences water stress, which can affect microbial activity and nutrient availability. This, in turn, leads to reduced nutrient cycling and decreased plant productivity. Conversely, increased moisture levels during the wet season may facilitate microbial metabolic processes, contributing to nutrient availability and soil fertility. Yet, the deep mechanisms underlying soil microbiome responses to seasonal fluctuations remain poorly understood [11, 15]. Consequently, elucidating these differences is imperative for optimizing soil health, enhancing ecosystem services, and mitigating climate change impacts.

The dry-hot valleys (DHVs) of southwest China present a unique and challenging environment for both microorganisms and plants. This region, which includes the valleys along the Jinsha River, Nu River, and Yuan River, covers more than 16,000 km^2^ and is characterized by an extended dry season lasting from November of the previous year to May of the current year, with low precipitation (about 50–150 mm, accounting for only 10% of the total annual precipitation) and high evaporation. During this period, the area experiences extreme aridity and high temperatures, creating a harsh environment for life. Microorganisms have been identified as pivotal biotic factors that facilitate plant adaptation to these harsh conditions [16]. Our investigations reveal that the valley exhibits a distinctive pattern of vertical vegetation distribution, characterized by a succession from savannas to forests as elevation increases. Above 3000 m a.s.l., the alpine zones boast lush forest vegetation and ample rainfall. The region spanning from 1600 to 3000 m a.s.l. serves as the transition zones between DHVs and the alpine zones. Within the transition zones, significant alterations in hydrothermal conditions and vegetation habitats occur, marking the interface between the two distinct ecological zones. Researchers have suggested local phenomena, including the foehn effect and mountain-valley breeze circulation, as significant contributors to this occurrence. Briefly, the foehn effect causes warming and drying of air on the leeward side of cross mountain wind. Particularly noteworthy is the pivotal role played by mountain-valley breeze circulation. In this process, diurnal temperature variations prompt mountain summits to warm more rapidly than valley floors during daylight hours, inducing lower air pressure atop the peaks. Consequently, airflow ascends, transporting moisture from the valley floors up the mountain slopes, thereby instigating the formation of valley breezes. In the evening, the mountain tops cool faster than the valleys, causing the airflow to sink and the cold air to descend along the mountain slopes, forming a mountain breeze. Over time, this process leads to increasing dryness in the valley floor, while moisture conditions remain suitable at higher elevations. Consequently, soil properties closely associated with water availability, such as pH, total nitrogen, total phosphorus, etc., do not exhibit significant seasonal differences in the low-elevation DHVs. These findings imply the importance of extending soil microbiome studies beyond low-elevation DHVs to encompass the entire valley ecosystem.

The intricate mechanisms governing community assembly represent a central challenge within microbial ecology [17–19]. Views of community assembly have traditionally been based on the contrasting perspectives of the deterministic niche paradigm and stochastic neutral models [20, 21]. Based on the niche theory, microbial community assembly is posited as a deterministic process influenced by abiotic factors (e.g., pH and temperature) and biotic factors (e.g., species interaction), reflecting diverse habitat preferences and microbial fitness. Conversely, the neutral theory postulates that microbial community assembly is governed by stochastic processes like birth, death, migration, speciation, and limited diffusion, assuming a stochastic equilibrium between taxon loss and acquisition [22]. Recent research into the microbial community assembly within DHVs remains sparse, leaving the underlying mechanisms poorly understood. Specifically, it remains uncertain whether soil microorganisms have developed seasonal community assembly mechanisms in response to environmental fluctuations within DHVs.

Previous studies have addressed the influence of various environmental factors on soil microbial composition, diversity, and function within DHVs, including desertification [23], land use change [13, 24, 25], vegetation [16, 26], and elevational gradients [27]. However, there exists a notable gap in research concerning the effects of seasonal dry and wet alternation [13, 14]. To address this gap, we examined the bacterial microbiome of 120 soil samples collected from DHVs, transition and alpine zones along the Jinsha River during both wet and dry seasons to compare the composition, structure, and function. We hypothesized that: i) microbes inhabiting DHVs have evolved efficient adaptive mechanisms to withstand persistent high temperature and drought, thereby minimizing the impact of seasonal dry and wet alternation on their diversity; ii) season-specific microbial clades contribute to responses of microbial taxa to seasonal dry and wet alternation; and iii) stochastic processes primarily govern the assembly of microbial communities in DHVs, whereas deterministic processes prevail in the transition and alpine zones due to substantial environmental disparities between dry and wet seasons.

## Methods

### Study area and sampling

**Fig. 1:**
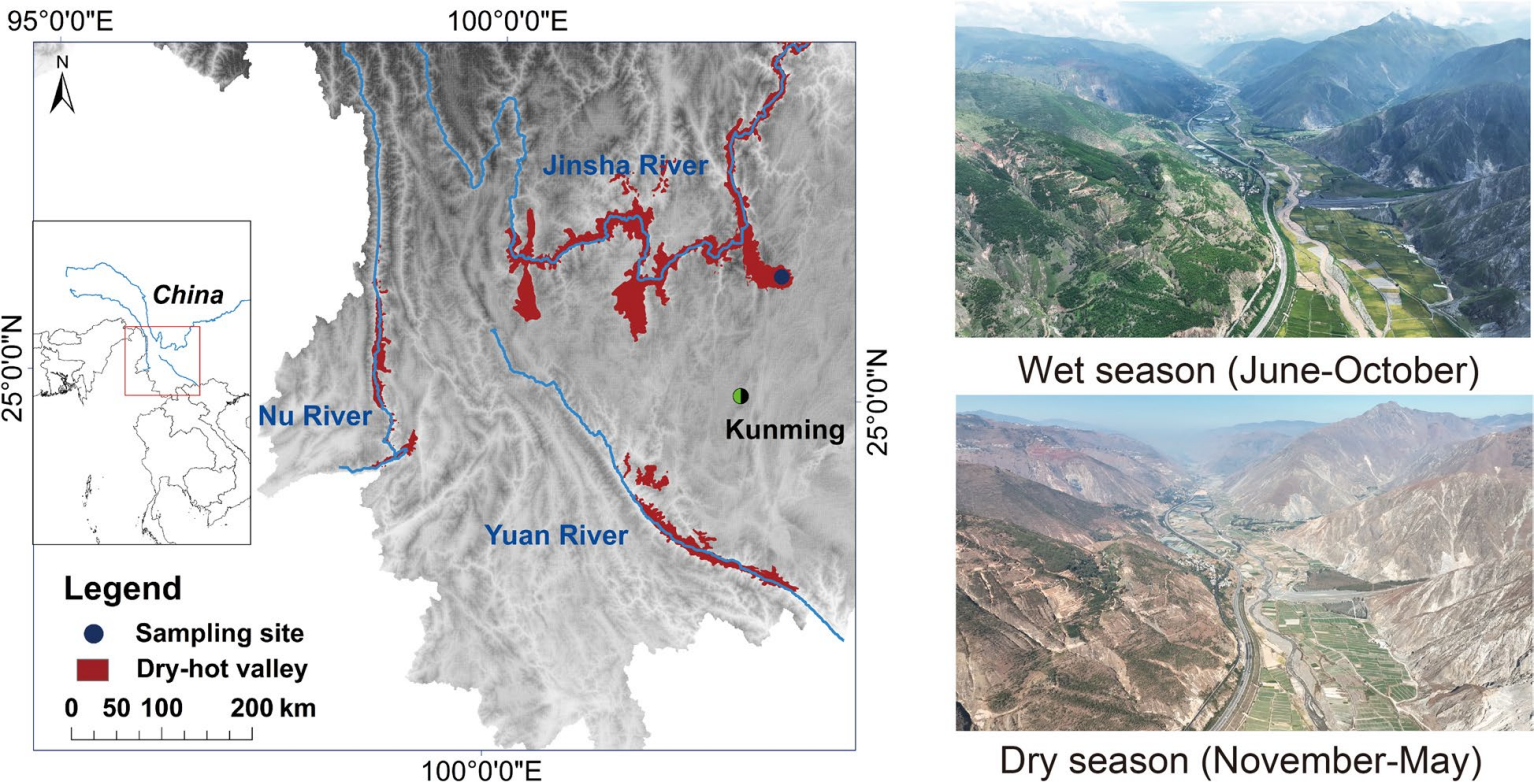
Overview of the study area. The photos on the right show the actual scenes of the study area during the dry and the wet seasons

This study was conducted in DHVs of the Xiaojiang River section, which serves as a first-class tributary of the Jinsha River (Fig. 1). The vegetation landscape below 1600 m above sea level (a.s.l.) on both sides of the river valley is characterized by a savanna-like ecosystem, with a hot and dry climate that is marked by a clear distinction between wet and dry seasons. To examine the differences in soil microbial community diversity between the dry and wet seasons in different elevations, we established sampling plots (20 m × 20 m) at three different elevations, based on long-term observation data. We recorded soil characteristics, vegetation composition, and meteorological information (Fig. 1 and Additional file 1: Supplementary Table S1). The sampling plots represent three distinct landscape types along the path of mountain-valley breeze circulation. Firstly, the DHV (~ 1100 m a.s.l.) is an area characterized by typical dry and hot conditions. The dominant vegetation in this region consists of savanna-like species, including *Dodonaea viscosa*, *Heteropogon contortus*, and *Agave sisalana*. Secondly, the high mountain area (Alpine zone, ~ 3000 m a.s.l.) is characterized by low temperatures and abundant rainfall. Planted trees, primarily *Pinus armandii*, are prevalent in this region. Lastly, the transition zone (~ 2000 m a.s.l.) lies between the two aforementioned areas. It is distinguished by sparse vegetation and is often surrounded by white cloud bands, which form as a result of the daytime valley breeze airflow rising.

In August 2019 and April 2020, soil samples were collected from three elevation gradients during the wet and dry seasons, respectively. To account for spatial heterogeneity within each sampling plot, ten soil cores were randomly collected from the upper 20 cm depth, and surface litter was meticulously removed. The soil cores were then pooled and homogenized to create a composite sample. A total of 20 composite soil samples were prepared from each sampling site for each season, resulting in a total of 120 composite samples for both seasons. The soils were sieved (< 2 mm) and separated into two portions: one was air-dried for one month and stored for soil biochemical analyses, and the other was immediately frozen at − 20 °C for molecular analyses. Soil pH was determined from the air-dried samples using a soil:solution ratio of 1:2.5. Soil total nitrogen (TN) concentrations were measured using an elemental analyser (Elementar Vario EL, Chengdu, China). Soil total phosphorus (TP) was measured by ICP-OES (Optima 8300, PerkinElmer, USA) as described by Yang et al. [28].

### DNA extraction, PCR amplification and amplicon sequencing

Genomic DNA was extracted from 0.5 g of each soil sample with the E.Z.N.A.® Soil DNA Kit (Omega Bio-Tek, Norcross, GA, USA) following the manufacturer’s instructions and stored at − 20 °C until further processing. The DNA extract was assessed on a 1% agarose gel, and DNA concentration and purity were determined with a NanoDrop 2000 UV–vis spectrophotometer (Thermo Scientific, Wilmington, DE, USA).

The hypervariable region V3-V4 of the bacterial 16S rRNA gene was amplified with the PCR primer pairs 338F (5'-ACTCCTACGGGAGGCAGCAG-3') and 806R (5'-GGACTACHVGGGTWTCTAAT-3') [29]. PCR amplification was performed as follows: initial denaturation at 95 °C for 3 min, followed by 27 cycles of denaturation at 95 °C for 30 s, annealing at 55 °C for 30 s, extension at 72 °C for 45 s, single extension at 72 °C for 10 min, and termination at 4 °C. The PCR mixtures contained 5 × TransStart FastPfu buffer (4 μL), 2.5 mM dNTPs (2 μL), forward primer (5 μM; 0.8 μL), reverse primer (5 μM; 0.8 μL), TransStart FastPfu DNA Polymerase (0.4 μL), bovine serum albumin (BSA; 0.2 μL), template DNA (10 ng), and up to 20 μL ddH_2_O. PCR reactions were performed in triplicate. The PCR products were extracted from 2% agarose gel and purified using the AxyPrep DNA Gel Extraction Kit (Axygen Biosciences, Union City, CA, USA) according to manufacturer’s instructions and were quantified using Quantus™ Fluorometer (Promega, Madison, WI, USA). Purified amplicons were pooled in equimolar concentrations and were paired-end sequenced on an Illumina MiSeq platform (Illumina, San Diego, CA, USA) by Majorbio Bio-Pharm Technology Co., Ltd. (Shanghai, China).

### Sequence processing

Raw fastq files were quality-filtered by Trimmomatic [30] and merged by FLASH [31] applying the following criteria: (i) Reads were truncated at any site receiving an average quality score < 20 over a 50-bp sliding window. (ii) Sequences with overlap longer than 10 bp were merged according to their overlap with mismatch of no more than 2 bp. (iii) Sequences of each sample were separated according to barcodes (exact matching) and primers (allowing a 2-nucleotide mismatch). Reads containing ambiguous bases were removed. Operational taxonomic units (OTUs) were clustered with a 97% similarity cutoff using UPARSE (version 7.0.1090) [32]. Mitochondrial and chlorophyll sequences were removed from the OTU table. The taxonomy of each 16S rRNA gene sequence was analyzed by the RDP classifier algorithm (version 2.11, http://rdp.cme.msu.edu/) against the Silva database (Release 138) [33], using a confidence threshold of 70% and implementation in QIIME (version 3.3.1) [34].

### Statistical analyses

Soil physicochemical properties were analyzed using the SPSS 17.0 software (SPSS Inc., Chicago, IL, USA). Significant differences among the means of different treatments were determined by Tukey’s multiple range tests after conducting tests of homogeneity for variances. Differences were considered statistically significant at the *P* < 0.05 level. To assess alpha-diversity, communities were rarified to the minimum sample sequence number (that is, 22647). The Sobs (community richness), Shannon (community diversity), and Good’s coverage index (community coverage) were calculated using QIIME (version 3.3.1) [34]. The significances of differences among treatments were compared using the Welch’s t-test. Beta-diversity was measured with the principal co-ordinate analysis (PCoA) using Bray–Curtis distances. ADONIS was carried out to evaluate group differences. The Welch’s t-test within STAMP [35] was used to identify bacterial phyla that showed significant differences in relative abundance between groups. *P*-values were adjusted for multiple comparisons using the Bonferroni method. Discriminant taxa were significantly retrieved by linear discriminant analysis (LDA) effect size (LEfSe) for soil bacterial communities between dry and wet seasons [36]. In order to explore the potential significance of stochastic processes in community assembly, a neutral community model (NCM) was used to examine the association between the detection frequency of OTUs and their relative abundance across the metacommunity [37]. Within this model, the parameter Nm serves as an estimate of dispersal among communities. Specifically, the Nm parameter determines the correlation between the frequency of occurrence and the regional relative abundance, where N represents the size of the metacommunity and m denotes the migration rate. The parameter R^2^ represents the overall goodness of fit to the neutral model. To calculate the 95% confidence intervals for all fitting statistics, bootstrapping was performed with 1000 bootstrap replicates. Moreover, the normalized stochasticity ratio (NST) was calculated to determine the contribution of the stochastic process to the microbial community assembly [21].

## Results

### Comparison of dry and wet season environmental factors at different elevations

During the dry and wet seasons, we documented variations in air temperature, air humidity, soil temperature, and soil moisture at various elevations (as shown in Additional file 1: Supplementary Table S2). Notably, both DHVs and the transition zones exhibited a distinct synoptic pattern in which drought and high temperatures persisted concurrently for the same period. Soil moisture in DHVs remained consistently low, hovering around 5% for an extended period, and was only higher than 6% in July and August. In contrast, the lowest value of 2.46% occurred in May, which was lower than that in the transition zones (6.41%) and alpine zones (8.1%), indicating extreme arid conditions. In both the wet and dry seasons in DHVs, there were no significant changes in soil pH, TN, TP, and N: P ratio, resulting in a statistically insignificant outcome (Additional file 1: Supplementary Table S3). In contrast, soil TN (*P* < 0.001) and TP (*P* < 0.001) in the transition zones were significantly lower in the dry season compared to the wet season, but soil pH remained unchanged (*P* = 0.987). Furthermore, in the alpine zones, there were no significant changes in TN and TP, except for a decrease in pH and N: P ratio in the dry season (Additional file 1: Supplementary Table S3).

### Soil bacterial diversity and composition

**Fig.2:**
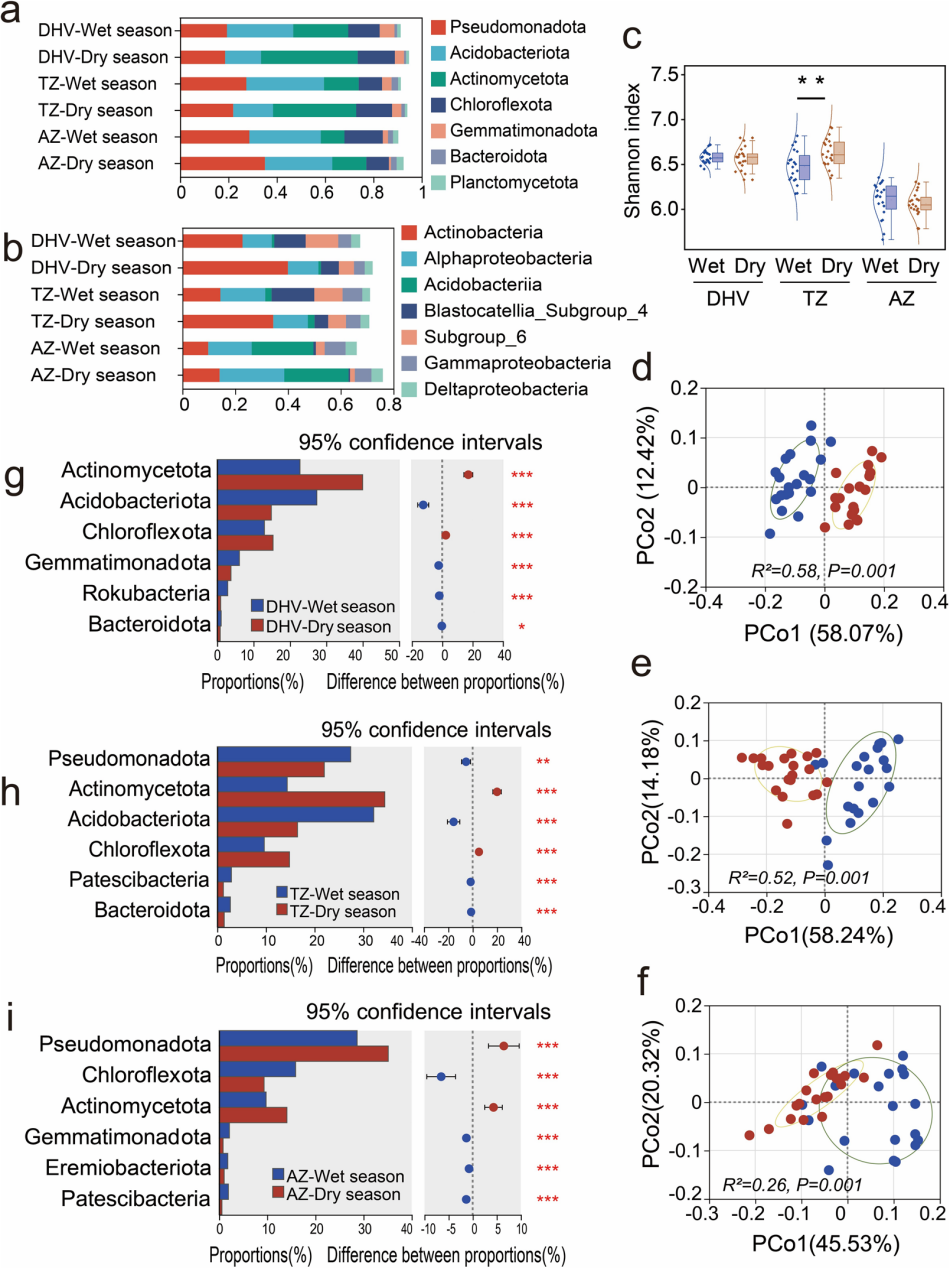
Effects of seasonal dry and wet alternation on soil bacterial community structure. The compositional shifts within the bacterial microbiome are shown at the (**a**) phylum and (**b**) class taxonomic levels, illustrating the ecological responses to climatic variation. Panel (**c**) presents a comparative analysis of the Shannon diversity index, which indicates the pronounced influence of seasonal dry and wet alternation in the transition zones (TZ), contrasting with those in the dry-hot valleys (DHVs) and alpine zones (AZ). Principal co-ordinate analysis (PCoA) and Adonis tests were performed at the phylum level, suggesting the distinct differences in (**d**) DHVs, (**e**) transition zones, and (**f**) alpine zones, respectively. An extended error bar plot identifying significant differences between the mean proportions of bacterial taxa between soil samples from (**g**) DHVs, (**h**) transition zones, and (**i**) alpine zones, corresponding to the wet (blue) and dry (brown) seasons, respectively. *, *P* < 0.05; **,* P* < 0.01; ***, *P* < 0.001

Bacterial community profiling yielded a total of 6,054,234 sequences ranging from 33,634 to 73,165 sequences per sample, which were obtained for the 120 soil samples. After subsampling each to the minimum number of sample sequences, 293,170 bacterial OTUs (approximately 2443 per sample) were identified, representing an average Good’s coverage of 95.82% (Additional file 2: Supplementary Figure S1). Our analysis of soil bacterial communities at different elevations revealed consistent alpha diversity in DHVs and the alpine zones during the dry and wet seasons. There were no statistically significant differences in community richness, diversity, or coverage. However, seasonal variations had a greater impact on community diversity in the transition zones (Fig. 2c), with higher diversity observed in the dry season compared to the wet season.

The dominant bacterial phyla in the soil were Pseudomonadota, Acidobacteriota, Actinomycetota, and Chloroflexota, collectively accounting for over 80% of the total abundance in both the dry and wet seasons (Fig. 2a). Additionally, we assessed the taxonomic composition at the class level, revealing significant variations among soil samples from different elevations and seasons. The dominant classes were Actinobacteria, Alphaproteobacteria, and Acidobacteriia (Fig. 2b). Moreover, we performed beta diversity analysis to examine the similarity or difference in community composition among samples. PCoA ordinations and Adonis tests demonstrated clear distinctions in bacterial community compositions between the dry and wet seasons for soils obtained from all three elevations (Fig. 2d, e, f).

### Specific microbial clades in soil bacteria community

We conducted a statistical analysis using Welch’s t-test with Bonferroni correction to compare the differences in soil bacterial phylum composition between the dry and wet seasons at various elevations. The relative abundance of Actinomycetota consistently exhibited higher levels in the dry season compared to the wet season across all soil samples at different elevations. In DHVs and the transition zones, the relative abundance of Chloroflexota was higher in the dry season compared to the wet season (Fig. 2g, h), while the opposite trend was observed in the alpine zones (Fig. 2i). Significant differences in the relative abundance of Pseudomonadota between the dry and wet seasons were primarily observed in the transition zones (*P* = 0.005, Fig. 2h) and alpine zones (*P* < 0.001, Fig. 2i). In the transition zone, the relative abundance of Pseudomonadota is higher in the wet season than in the dry season. On the contrary, in the alpine zone, the relative abundance of Pseudomonadota is lower in the wet season compared to the dry season.

**Fig.3:**
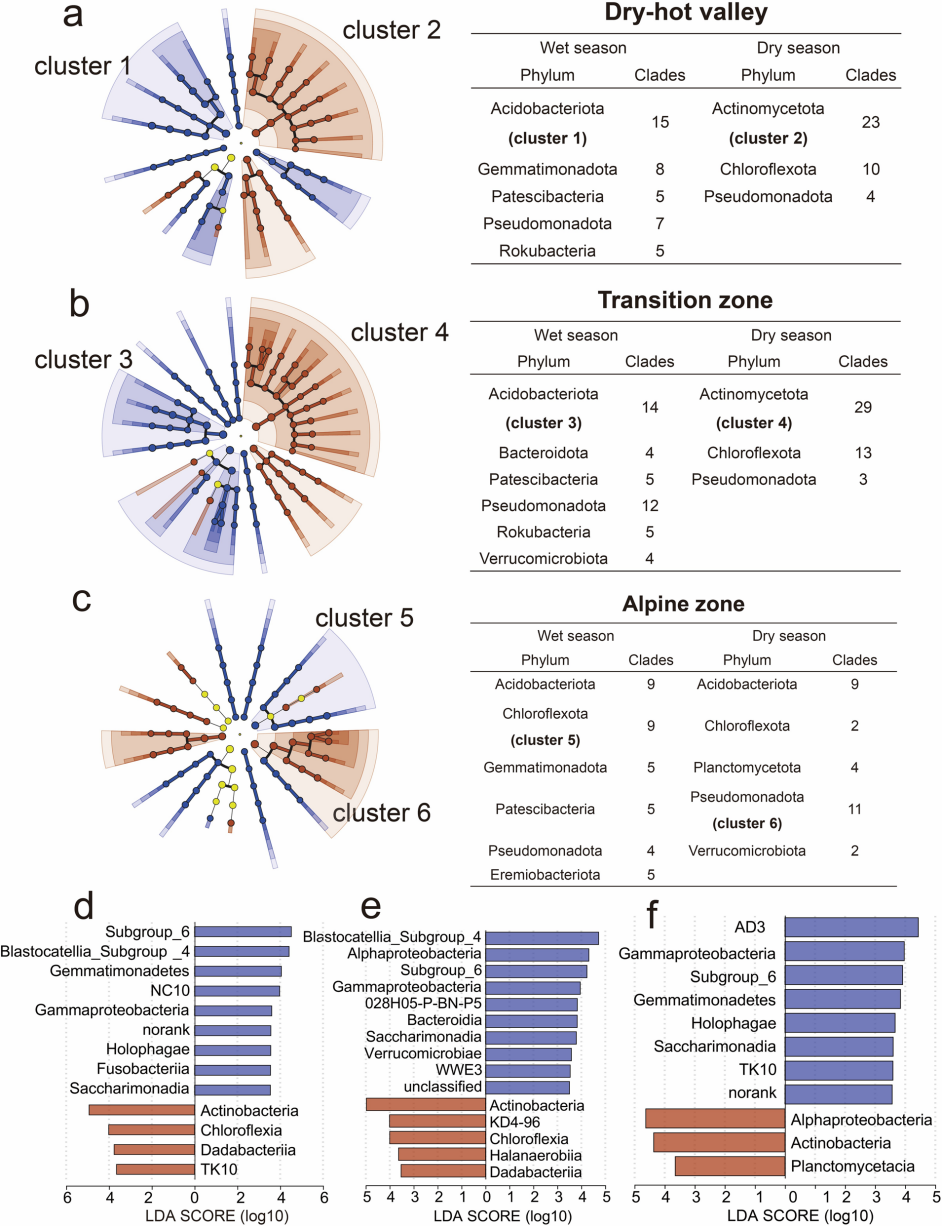
Discriminant taxa significantly retrieved by linear discriminant analysis (LDA) effect size (LEfSe) for soil bacterial communities between dry and wet seasons. The cladogram indicates the taxonomic representation of statistically consistent differences between soil samples from dry (brown) and wet (blue) seasons in (**a**) dry-hot valleys (DHVs), (**b**) transition, and (**c**) alpine zones, respectively. Discriminant clades are documented on the right. Histogram of the LDA score determined for differentially abundant taxa (class level) with cut-off LDA score > 3.5, *p* < 0.05 (**d**-**f**). Negative LDA score (brown) highlight the enriched taxa in soil samples from dry seasons and positive LDA score (blue) are abundant taxa in soil samples from wet seasons

To further identify microorganisms that can effectively differentiate between the dry and wet seasons at different elevations, we employed LEfSe to visualize the distribution of various clades at the phylum to genus levels in soil samples (Fig. 3).

During the dry season, we observed similarities in microbial clades between soils from DHVs (Fig. 3a) and the transition zones (Fig. 3b), including the phyla Actinomycetota, Chloroflexota, and Pseudomonadota. However, we found a predominance of phyla Acidobacteriota (9), Planctomycetota (4), and Verrucomicrobiota (2) from the alpine zones (Fig. 3c). Additionally, we observed a significant decrease in the relative abundance of Chloroflexota and an increase in Pseudomonadota in the alpine zones, as determined by the same screening criteria (LDA score > 3.5, *p* < 0.05). In contrast to the dry season, soil samples from the wet season exhibited a higher diversity of microbial clades, including the presence of specific clades such as Acidobacteriota, Patescibacteria, and Pseudomonadota. Verrucomicrobiota (4) was exclusively detected in the transition zones, while Eremiobacteriota (5) was specifically found in the alpine zones. Finally, histograms of the LDA scores (LDA score > 3.5, *p* < 0.05) implied bacterial clades (class level) showing statistically significant and biologically consistent differences between the dry and wet seasons at different elevations (Fig. 3d-f).

### Environmental factors influencing community structure

**Fig.4:**
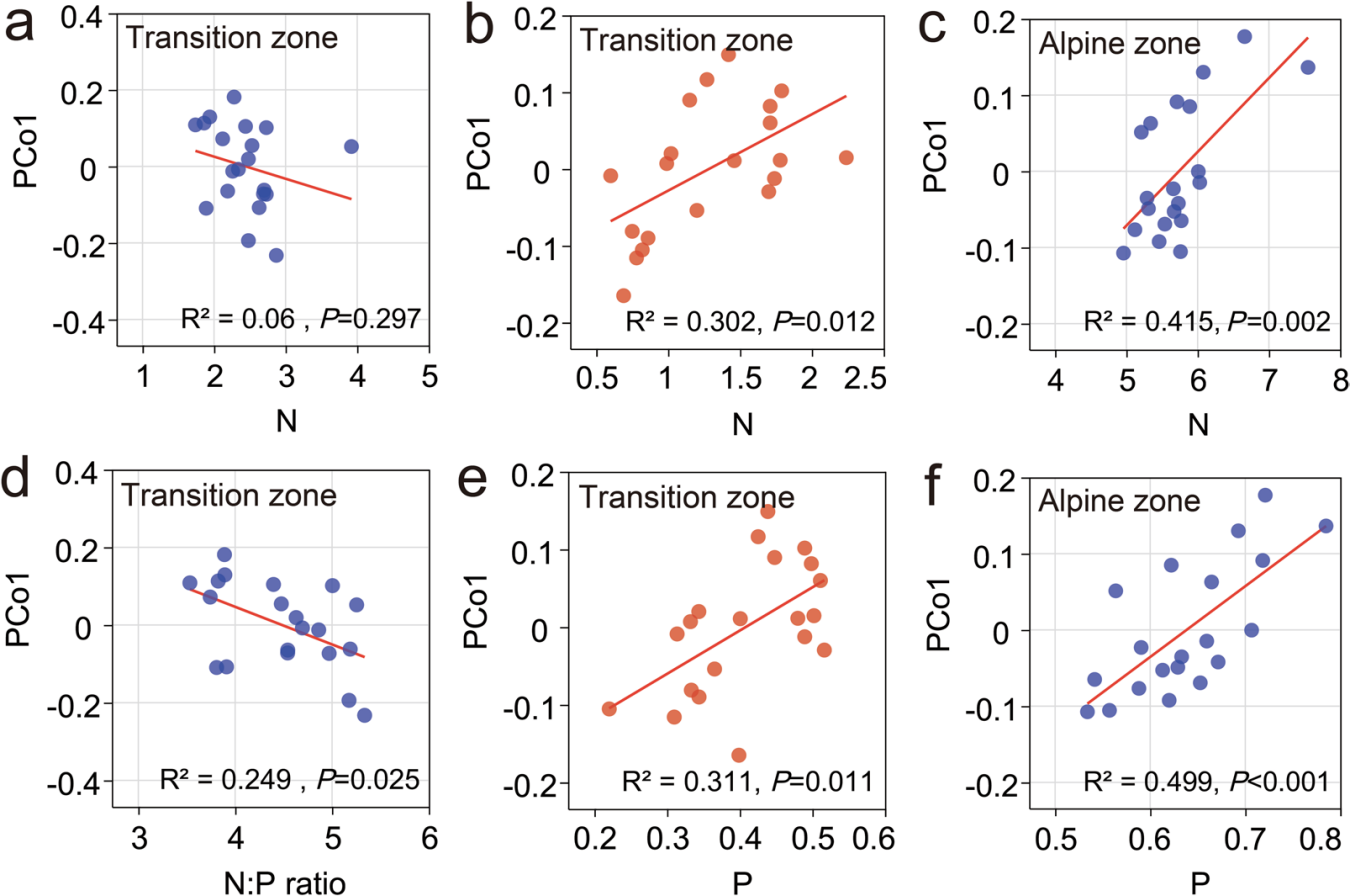
Linear regression analysis elucidating the relationship between nutrient availability and phylum-level beta diversity during the dry (brown) and wet (blue) seasons, respectively

We conducted a linear regression analysis utilizing the first principal axis (PCo1) of PCoA to elucidate the relationship between environmental variables and beta-diversity at the phylum level. Out of the 24 potential combinations examined, only five exhibited statistical significance (Fig. 4). Notably, nutrient availability in the transition zones exerted an impact on PCo1 during both the dry season (N and P) and the wet season (N:P ratio), whereas in the alpine zones, nutrient availability influenced PCo1 only during the wet season (N and P). Remarkably, bacterial communities displayed no significant correlation with environmental factors, with the exception of N and P in the transition zones during the dry season.

**Fig.5:**
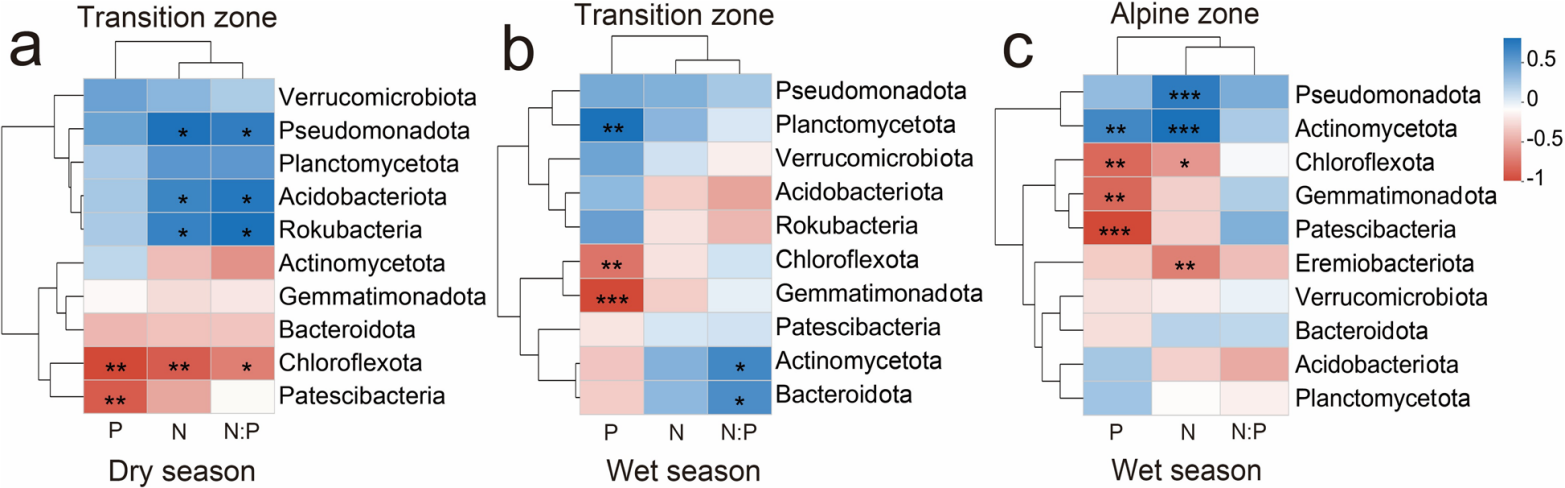
Correlation heatmap between dominant phyla of soil bacterial community and N, P and N:P ratio in transition and alpine zones during the dry and wet seasons. *, *P* < 0.05; **, *P* < 0.01; ***, *P* < 0.001

Furthermore, the correlation heatmap revealed the correlation coefficients and statistically significant correlations between the dominant phyla (top 10) of soil bacterial communities and environmental factors (N, P, and N:P ratio) in the transition zones (Fig. 5a, b) and alpine zones (Fig. 5c), respectively. Particularly, soil P and N:P ratio exhibited substantial effects on the relative abundance of Actinomycetota, Bacteroidota, Chloroflexota, Gemmatimonadota, and Planctomycetota in the wet season within the transition zones (Fig. 5b).

### Ecological assembly of bacterial community

**Fig.6:**
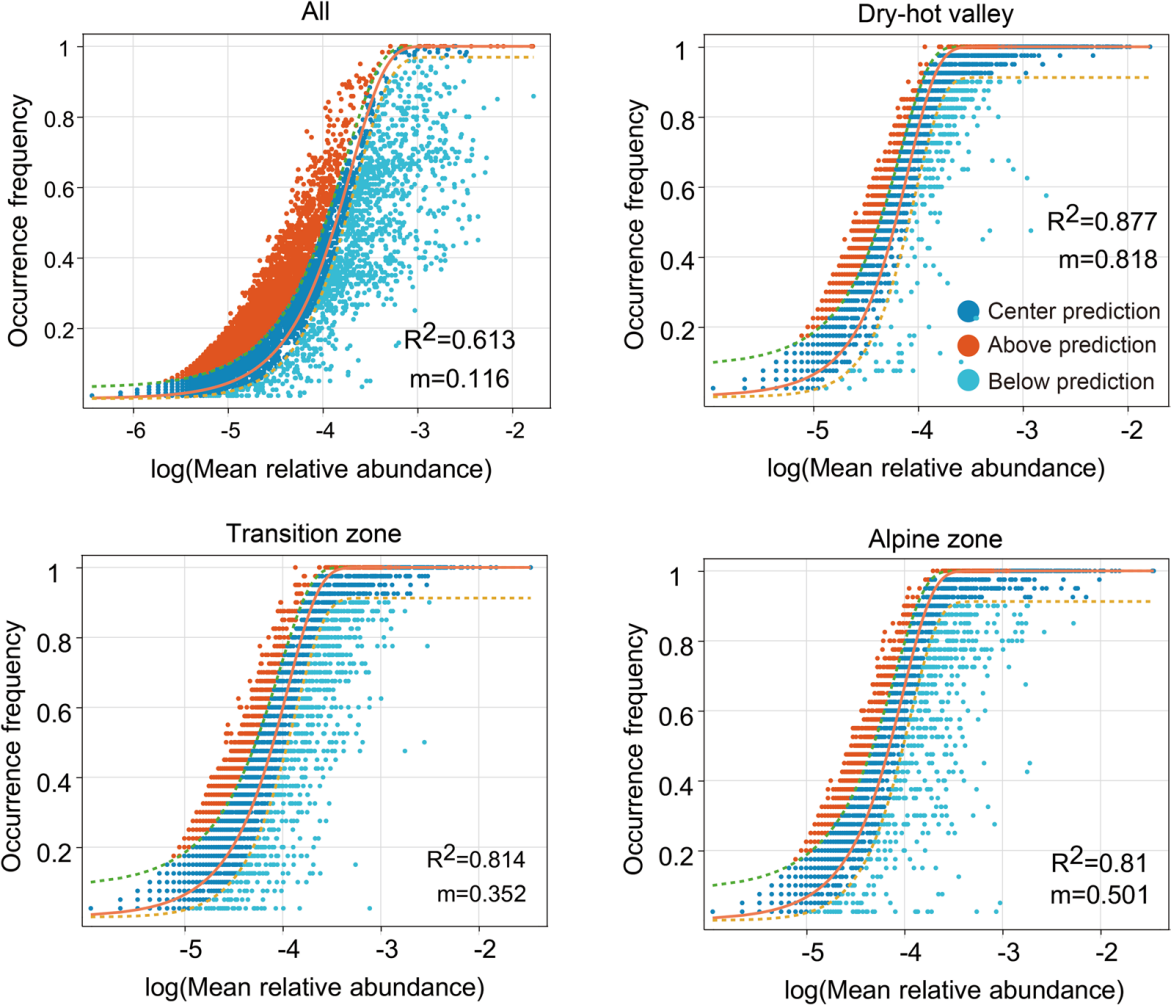
Fit of the neutral community model (NCM) of bacterial community assembly. The solid orange line represents the best fit to the model, with dashed green and yellow lines indicating the 95% confidence intervals. OTUs deviate from the model’s predictions, either occurring more or less frequently, are highlighting in distinct colors. The R^2^ indicates the goodness of fit to the model, with a higher value denoting a better fit. The migration rate (m) represents the dispersal ability of species within the community. A smaller m-value suggests restricted dispersal and a more localized community, while a higher m-value indicates less restricted dispersal and a more interconnected community

A comprehensive investigation was conducted on soil microbial communities at varying elevations and seasons (Fig. 6 and Additional file 2: Supplementary Figure S2), uncovering the influence of stochastic processes on the assembly of soil bacterial communities throughout the entire valley (R^2^ = 0.613). Notably, within this geographical region, microorganisms face high diffusion restrictions, with a migration rate (m) of 0.116. In-depth analysis of soil bacterial communities at various elevations revealed that DHVs exhibited the high fit to the NCM at 0.877 and had low diffusion restrictions for its microorganisms (m = 0.818). Among the three elevations, the transition zones between DHVs and the alpine zones exhibited the highest level of dispersal limitation for its microorganisms (m = 0.352), indicating greater restrictions on their dispersal. Given that the number of sequences in each sample was consistent (*N* = 22,647), the estimates of intercommunity dispersal, which represent the movement of microorganisms between different communities, were highest in DHVs (Nm = 18,526), followed by the alpine zones (Nm = 11,342), and lowest in the transition zones (Nm = 7,964). Furthermore, an analysis of the disparity in migration rates at different elevations during the dry and wet seasons was undertaken. Intriguingly, migration rates in both DHVs and the alpine zones were found to be higher in the dry season compared to the wet season, suggesting reduced diffusion constraints. In contrast, this pattern was reversed in the transition zones. Overall, NST analysis again quantitatively revealed that stochastic processes played a crucial role in shaping microbial community assembly, with all samples exhibiting an NST value exceeding 50% (Additional file 2: Supplementary Figure S3).

## Discussion

The hydrothermal conditions in the Jinsha River valley exhibit significant variations at different elevations. The lower elevation areas exhibit a persistently arid and high-temperature climate, characterized by minimal fluctuations in both moisture and temperature throughout the dry and wet seasons. This stands in stark contrast to the high elevation forests and the transition zones between them. Numerous studies have highlighted the crucial role of soil microorganisms in the ecological restoration of the dry-hot valley. We propose that soil microorganisms in the low elevation DHVs have developed tolerance mechanisms to effectively cope with seasonal dry–wet alternation and even the extreme dry and hot environments they encounter during long-term adaptation processes [4, 6, 38, 39]. As expected, the bacterial communities within DHVs exhibited strong stability to seasonal dry and wet alternation (Fig. 2). Our results indicated that the core bacterial taxa, which accounted for over 80% of the community, remained consistent. These taxa were prominently represented by Pseudomonadota, Acidobacteriota, Actinomycetota, and Chloroflexota (Fig. 2a). However, these findings diverge from observations in other regions of the DHVs in southwest China [13, 23–25, 40]. It may suggest that although the arid and hot environment exerts a selective pressure on the soil microbial community [41, 42], the composition of the core microbial community is intricately linked to soil properties, vegetation diversity, and other environmental factors [43, 44]. Within DHVs and the transition zones, the relative abundance of Actinomycetota and Chloroflexota was increased during the dry season, in contrast to Acidobacteriota, which displayed an opposite trend (Fig. 2g, h). These microbial dynamics are posited to critically influence the microbiome’s regulation to seasonal moisture fluctuations [45]. Notably, Actinomycetota exhibit robustness to environmental stress, including drought, and are instrumental in soil functionality under challenging conditions [10, 46, 47]. Their symbiotic relationships with plant roots further bolster drought tolerance by facilitating phytohormone production and enhanced nutrient acquisition [10, 42, 48]. In addition, Chloroflexota, a phylum adapted to oligotrophic conditions, demonstrates the capacity to prosper in resource-limited environments [49–51]. Our findings align with the results [45] in suggesting that Chloroflexota may interact with other soil microorganisms, thereby shaping community structure and function. Taken together, these results underscore the crucial role of Actinomycetota and Chloroflexota in promoting soil resilience and ecosystem stability under the extreme conditions found in DHVs [12].

Seasonal fluctuations in soil temperature, moisture, and nutrient levels can significantly influence belowground microbial communities. However, these seasonal variations may not be the primary driver of microbiota diversity, as it is influenced more by taxon-specific gene expression [8]. Our research revealed that soil bacterial communities at various elevations maintained consistent richness, diversity, and coverage throughout both dry and wet seasons, except within the transition zones (see Fig. 2c). This observation suggests that the regulation of season-specific microbial clades may contribute to the stability of microbial community structure. For example, our study identified an increase in the number of clades associated with Acidobacteriota and Patescibacteria within DHVs and the transition zones during the wet season. The number of these clades subsequently decreased following a prolonged dry period. In contrast, the number of clades within specific bacterial phyla increased (Fig. 3a-c). Interestingly, some clades exhibited complex regulatory patterns, such as those related to Pseudomonadota.

Within the lower elevation DHVs, clades associated with Actinomycetota were more abundant during dry seasons compared to wet seasons. Previous studies have highlighted the pivotal role of Actinomycetota in responding to drought stress, including their ability to maintain activity and enter a dormant state under dry conditions. Santos-Medellín et al. [52] proposed that prolonged drought leads to a significant enrichment of Actinomycetota in the rice rhizosphere microbiome, with their abundance declining upon recovery. Therefore, we hypothesize that Actinomycetota and its related clades play a regulatory role in response to changes in water availability, enhancing the functionality of soil bacterial communities during seasonal dry–wet cycles. Acidobacteriota, a widely distributed phylum in diverse ecosystems [53, 54], demonstrates a remarkable regulatory capacity and adaptive mechanisms to thrive in complex environmental conditions. Kalam et al. [55] conducted a comprehensive review of current research on Acidobacteriota in soil, highlighting its significant ecological importance. Our findings revealed a notable decline in the relative abundance of Acidobacteriota-related microbial clades in DHVs and the transition zones during dry seasons, with a resurgence of relative abundance observed during wet seasons when water availability increased (Fig. 3a, b). Studies suggest that Acidobacteriota may possess specific genes facilitating survival and competitive colonization in the rhizosphere, fostering beneficial interactions with plants [56, 57]. In contrast, fluctuations in the relative abundance of Pseudomonadota-associated clades across different sampling areas did not exhibit consistent patterns during the transition from wet to dry seasons (Fig. 3a-c), indicating diverse regulatory strategies employed by Pseudomonadota-related microbial clades in response to seasonal variations [9, 47, 58]. Notably, seasonal fluctuations in the alpine soil microbial community, while evident, are relatively minor compared to DHVs and the transition zones, possibly due to the limited impact of moisture and temperature changes in alpine regions. This suggests that soil bacterial communities in alpine zones maintain stability under the mild seasonal fluctuations of wet and dry conditions. Thus, our results reveal that soil microbial communities undergo succession in response to selective pressures imposed by seasonal dry–wet alternations. Adaptive evolution may result in the emergence of novel traits or genetic variants that confer fitness advantages under specific moisture regimes [59–61]. Genetic adaptation to fluctuating environmental conditions contributes to the resilience and stability of soil microbial aggregates over time.

Recent advancements in microbial community assembly research have illuminated our understanding of these intricate ecological dynamics. However, the impact of seasonal dry and wet alternation on community assembly within unique savanna-like dry-hot valley ecosystems remains poorly characterized across spatial and temporal scales. This study investigates the influence of environmental factors on microbial communities in these distinct ecosystems. Our results indicate a minimal effect of seasonal dry and wet alternation in valley regions, but varying degrees of alteration in transition and alpine zones. This observation led us to hypothesize that soil microbial community assembly in dry-hot valley ecosystems is predominantly driven by stochastic processes, in contrast to the deterministic processes observed in the transition and alpine zones. Employing the neutral community model (NCM), our analysis emphasizes the dominance of stochastic mechanisms in microbial community assembly within valley ecosystems, particularly in savanna-like dry-hot valleys (Fig. 6). This finding partially supports our hypothesis and suggests that stochastic processes play a significant role in shaping microbial communities in these ecosystems. However, the influence of stochastic processes extends to the transition and alpine zones as well. Our findings challenge previous assumptions about the primary influence of environmental factors, such as seasonal dry and wet alternation, on shaping microbial communities across all ecosystem types. Soil pH, a crucial abiotic factor affecting soil microbial communities, could mediate the balance between stochastic and deterministic assembly of bacteria [62]. In our study, soil pH in DHVs and the transition zones did not vary significantly between dry and wet seasons, except in the alpine zones (Additional file 1: Supplementary Table S3). Nevertheless, the potential role of deterministic processes in dry and hot valley regions cannot be discounted. It is well-established that deterministic and stochastic processes coexist in regulating ecological community assembly [20, 21, 63]. Moreover, Thompson et al. [64] proposed that narrow abiotic niche curves lead to strong species responses to environmental variation (deterministic processes), while broad or flat curves result in weak or absent responses (stochastic processes). Our study suggests that the dry-hot valleys may exhibit characteristics that favor stochastic assembly, potentially influenced by reduced environmental variability or increased dispersal rates [65]. Increased dispersal rates can lead to greater mixing of species and a more homogeneous community composition. Higher dispersal rates directly enhance the immigration component. For instance, if two species arrive simultaneously and compete randomly, the outcome of this interaction is a chance event that can shape the community in a stochastic manner [20, 37].

## Conclusions

**Fig.7:**
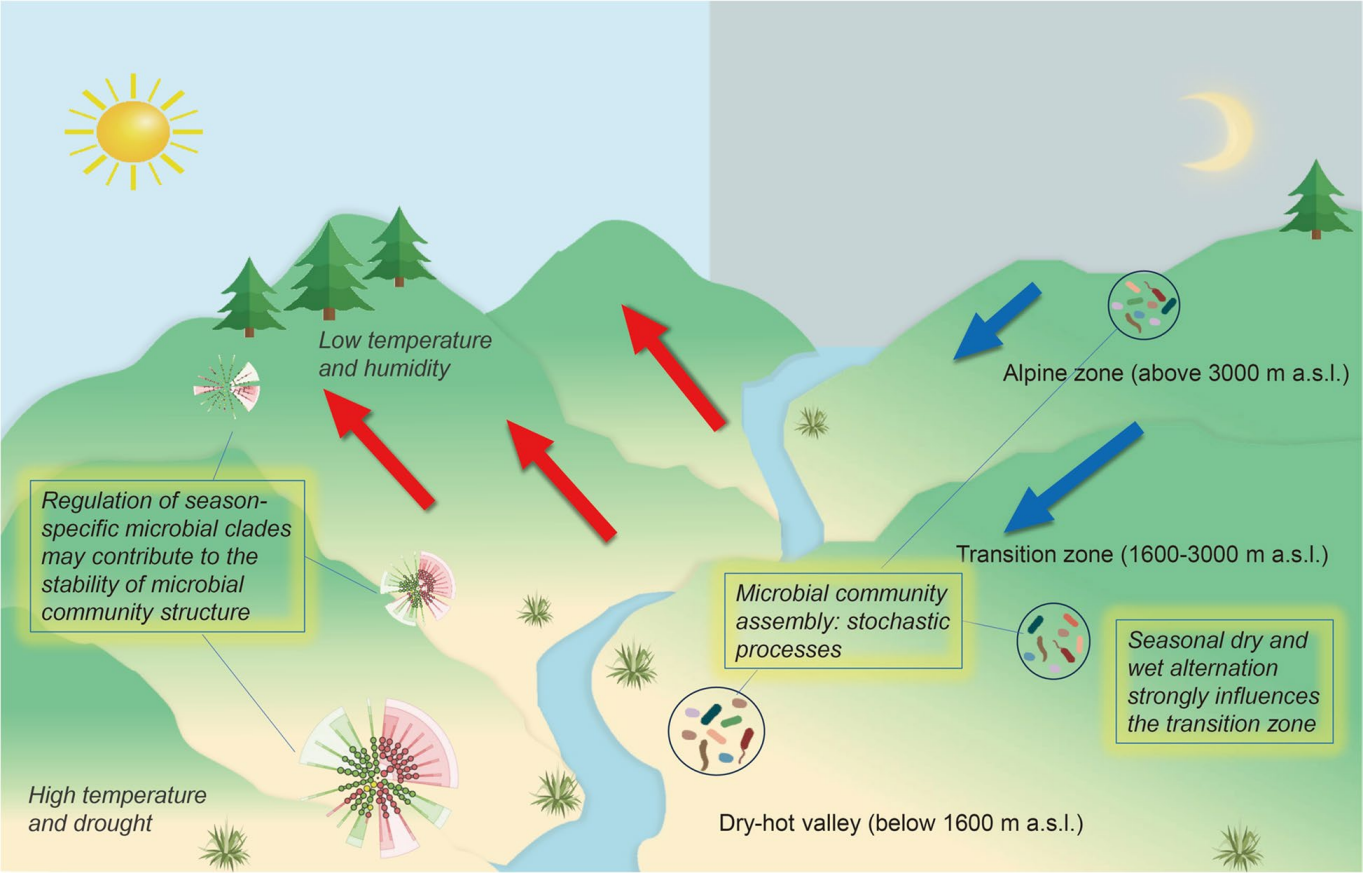
Schematic diagram of the seasonal dynamics of soil microbiome responding to dry–wet alternation along the Jinsha River Dry-hot Valley. The distinctive ecological characteristics of the dry-hot valleys are defined by their attributes: aridity and high temperatures. Seasonal dry and wet alternation leads to this phenomenon being prominent. This unique climate exhibits a clear vertical distribution, with elevation playing a pivotal role in shaping the environment and vegetation. As one descends from the mountain peaks, the landscape transforms from lush evergreen or coniferous forests into savanna or scrubland. The formation of this ecological landscape is a fascinating subject of scientific study, with a multitude of factors contributing to its uniqueness. Among the various influences, the daily mountain-valley breeze circulation stands out as a prominent and consistent phenomenon. During the day, rapid warming at the mountaintop triggers a decrease in air pressure, inducing moisture-laden valley breezes (indicated by red arrows) that ascend from the valley floor. Conversely, at night, the swift drop in temperature at higher altitudes generates descending air currents, forming mountain breezes (depicted by blue arrows). We propose that the soil microorganisms native to the valley floors of dry-hot valleys (DHVs) have likely evolved specific mechanisms to adapt to these conditions over extended periods. For instance, microbial community assembly, dominated by stochastic processes, may play a significant role. Additionally, the involvement of season-specific microbial clades and cascades in the adaptation process cannot be underestimated, as they potentially influence and are influenced by the unique environmental factors. Of particular interest are the transition zones (1600–3000 m a.s.l.), situated between the DHVs (below 1600 m a.s.l.) and the alpine zones (above 3000 m a.s.l.), where moisture and temperature conditions become favorable. This intermediate area exhibits relatively higher microbial community and functional diversity compared to the extreme environments of the valley floors and mountain peaks. Therefore, this zone presents itself as a strategic focus for ecological restoration efforts, leveraging the intricate interplay between plant and microbial life

Our study demonstrates the remarkable influence of seasonal dry and wet alternation on bacterial community diversity within transition zones, surpassing its impact in both DHVs and alpine zones. Notably, we identified season-specific microbial clades across all sampling areas, highlighting their responsiveness to dynamic environmental conditions. Stochastic processes emerged as the dominant drivers of soil bacterial community assembly in all three zones. Here, we suggest a schematic diagram illustrating the seasonal dynamics of the soil microbiome in response to dry–wet alternation along the Jinsha River Dry-hot Valley (Fig. 7).

In conclusion, these findings hold implications for developing strategies to optimize soil health, enhance ecosystem services, and mitigate the effects of climate change in these distinct regions: DHVs, transition zones, and alpine zones.

## Supplementary Information

Additional file 1: Supplementary Table S1. The following information is provided on the location, soil types, and plant species of the sampling plots. * Classification and codes for Chinese soil (GB/T 17296-2009). Supplementary Table S2. The meteorological conditions, soil temperature and moisture data for the study sites were recorded from June 2019 to May 2020. The wet season is defined as the period from June to October, while the dry season is defined as the period from November to May. Supplementary Table S3. Seasonal differences in soil pH, total nitrogen, and phosphorus content between the wet and dry seasons. Different letters in the same column represent statistical significance between groups (means ± SE, *n*=20) at *P* < 0.05 according to Tukey’s multiple range tests. TN, total nitrogen; TP, total phosphorus.

Additional file 2: Supplementary Figure S1. Rarefaction curves of community richness (Sobs index), diversity (Shannon index), and coverage (Good’s coverage index) on OTU level across all soil bacterial communities. Supplementary Figure S2. Fit of the neutral community model (NCM) of bacterial community assembly. The solid orange line represents the best fit to the model, with dashed yellow and green lines indicating the 95% confidence intervals. OTUs deviate from the model’s predictions, either occurring more or less frequently, are highlighting in distinct colors. The R^2^ indicates the goodness of fit to the model, with a higher value denoting a better fit. The migration rate (m) represents the dispersal ability of species within the community. A smaller m-value suggests restricted dispersal and a more localized community, while a higher m-value indicates less restricted dispersal and a more interconnected community. Supplementary Figure S3. Comparison of the normalized stochasticity ratio (NST) between soil bacterial communities at different elevations during the wet (blue) and dry (brown) seasons. The NST was calculated based on Jaccard using the null model algorithm PF. In this study, 50% was used as the boundary between deterministic (<50%) and stochastic (>50%) processes. *, *P *< 0.05; **, *P *< 0.01; ***, *P *< 0.001.

## Data Availability

The data that support the findings of this study are available in the GitHub repository: https://github.com/Haoimde/Seasonal-Dynamics-of-Soil-Microbiome-in-Response-to-Dry–Wet-Alternation. The raw soil bacterial 16S rRNA sequencing data was deposited into the NCBI Sequence Read Archive (SRA) database under Accession Number: PRJNA1114417.
