# Supplementary material for: Seasonal dynamics of soil microbiome in response to dry–wet alternation along the Jinsha River Dry-hot Valley
Source: BMC Microbiol. 2024 Nov 25;24:496. doi: 10.1186/s12866-024-03662-1 (PMC11587743; doi:10.1186/s12866-024-03662-1)
Supplement: Supplementary file 1 — Additional file 1: Supplementary Table S1. The following information is provided on the location, soil types, and plant species of the sampling plots. * Classification and codes for Chinese soil (GB/T 17296-2009). Supplementary Table S2. The meteorological conditions, soil temperature and moisture data for the study sites were recorded from June 2019 to May 2020. The wet season is defined as the period from June to October, while the dry season is defined as the period from November to May. Supplementary Table S3. Seasonal differences in soil pH, total nitrogen, and phosphorus content between the wet and dry seasons. Different letters in the same column represent statistical significance between groups (means ± SE, n=20) at P < 0.05 according to Tukey’s multiple range tests. TN, total nitrogen; TP, total phosphorus. [file 12866_2024_3662_MOESM1_ESM.docx]

**Supplementary Information**

**Additional file 1**

**Supplementary Table S1** The following information is provided on the location, soil types, and plant species of the sampling plots.

| Plot | Elevation (m) | Latitude | Longitude | Soil type * | Plant species |
| --- | --- | --- | --- | --- | --- |
| Dry-hot valley | ~1100 | 26°15′52′′ N | 103°6′ 54′′ E | Alluvial soil | *Leucaena leucocephala*, *Eucalyptus robusta, Calotropis gigantean, Dodonaea viscosa, Barleria cristata, Sophora davidii, Agave sisalana, Heteropogon contortus* |
| Transition zone | ~2000 | 26°14′32′′ N | 103°4′ 34′′ E | Skeletol soil | *Glochidion eriocarpum*, *Artemisia caruifolia, Cupressus funebris, Oxyria sinensis, Acacia dealbata, Buddleja officinalis, Populus cathayana, Pteridium aquilinum var. latiusculum* |
| Alpine zone | ~3000 | 26°15′55′′ N | 103°0′ 40′′ E | Yellow-brown earth | *Abies fabri, Pinus yunnanensis, Rhododendron macrophyllum, Quercus myrsinifolia, Pinus armandi, Viburnum cylindricum* |

* Classification and codes for Chinese soil (GB/T 17296-2009)

**Supplementary Table S2** The meteorological conditions, soil temperature and moisture data for the study sites were recorded from June 2019 to May 2020. The wet season is defined as the period from June to October, while the dry season is defined as the period from November to May.

|  | | Dry-hot valley  (~1100 m a.s.l.) | Transition zone  (~2000 m a.s.l.) | Alpine zone  (~3000 m a.s.l.) |
| --- | --- | --- | --- | --- |
| Air temperature (℃) | Annual temperature | 21.19 | 15.88 | 8.97 |
|  | Maximum of monthly mean temperature | 26.39 (June) | 23.4 (June) | 14.4 (June) |
|  | Minimum of monthly mean temperature | 14.31 (December) | 9.19 (December) | 3.08 (December) |
|  | Sample time (monthly mean temperature) | 24.45 (August)  21.85 (April) | 20.76 (August)  15.4 (April) | 13.41 (August)  8.52 (April) |
| Air humidity (%) | Annual humidity | 53.9 | 66.4 | 74.39 |
|  | Maximum of monthly mean humidity | 67.04 (September) | 84.97 (July) | 92.89 (September) |
|  | Minimum of monthly mean humidity | 38.34 (March) | 48.49 (March) | 51.66 (March) |
|  | Sampling time (monthly mean humidity) | 56.26 (August)  48.12 (April) | 71.11 (August)  57.88 (April) | 81.46 (August)  70.04 (April) |
| Soil temperature  (Depth 20 cm, ℃) | Annual temperature | 23.88 | 18.36 | 9.22 |
|  | Maximum of monthly mean temperature | 30.2 (May) | 26.2 (May) | 14.53 (June) |
|  | Minimum of monthly mean temperature | 17.37 (December) | 11.04 (February) | 1.94 (Janury) |
|  | Sampling time (monthly mean temperature) | 25.97 (August)  25.52 (April) | 23.01 (August)  18.94 (April) | 14.4 (August)  9.84 (April) |
| Soil moisture  (Depth 20 cm, %) | Annual moisture | 4.87 | 8.83 | 9.87 |
|  | Maximum of monthly mean moisture | 8.25 (July) | 13.5 (July) | 13.29 (February) |
|  | Minimum of monthly mean moisture | 2.46 (May) | 6.41 (May) | 8.1 (May) |
|  | Sampling time (monthly mean moisture) | 7.3 (August)  3.71 (April) | 11.75 (August)  7.44 (April) | 9 (August)  11.12 (April) |

**Supplementary Table S3**  **Seasonal differences in soil pH, total nitrogen, and phosphorus content between the wet and dry seasons.**

| Plot | pH | TN (g kg^-1^) | TP (g kg^-1^) | N:P ratio |
| --- | --- | --- | --- | --- |
|  | Wet season | | |  |
| Dry-hot valley (~1100 m a.s.l.) | 8.51±0.02a | 2.86±0.11bc | 0.87±0.02a | 3.28±0.08d |
| Transition zone (~2000 m a.s.l.) | 6.99±0.04b | 2.44±0.11c | 0.54±0.02c | 4.48±0.13c |
| Alpine zone (~3000 m a.s.l.) | 5.26±0.08c | 5.74±0.13a | 0.64±0.01b | 9.02±0.16a |
|  | Dry season | | |  |
| Dry-hot valley (~1100 m a.s.l.) | 8.41±0.03a | 2.93±0.1b | 0.87±0.02a | 3.35±0.04d |
| Transition zone (~2000 m a.s.l.) | 7.04±0.09b | 1.28±0.1d | 0.41±0.02d | 3.09±0.15d |
| Alpine zone (~3000 m a.s.l.) | 4.84±0.03d | 5.67±0.12a | 0.7±0.01b | 8.13±0.17b |

Different letters in the same column represent statistical significance between groups (means ± SE, n=20) at *P* < 0.05 according to Tukey’s multiple range tests. TN, total nitrogen; TP, total phosphorus.
